# Equivalence model: A new graphical model for causal inference

**DOI:** 10.4178/epih.e2020024

**Published:** 2020-04-09

**Authors:** Jalal Poorolajal

**Affiliations:** 1 Department of Epidemiology, School of Public Health, Hamadan University of Medical Sciences, Hamadan, Iran; 2 Modeling of Noncommunicable Diseases Research Center, School of Public Health, Hamadan University of Medical Sciences, Hamadan, Iran

**Keywords:** Causality, Risk factors, Protective factors, Epidemiologic methods

## Abstract

Although several causal models relevant to epidemiology have been proposed, a key question that has remained unanswered is why some people at high-risk for a particular disease do not develop the disease while some people at low-risk do develop it. The equivalence model, proposed herein, addresses this dilemma. The equivalence model provides a graphical description of the overall effect of risk and protective factors at the individual level. Risk factors facilitate the occurrence of the outcome (the development of disease), whereas protective factors inhibit that occurrence. The equivalence model explains how the overall effect relates to the occurrence of the outcome. When a balance exists between risk and protective factors, neither can overcome the other; therefore, the outcome will not occur. Similarly, the outcome will not occur when the units of the risk factor(s) are less than or equal to the units of the protective factor(s). In contrast, the outcome will occur when the units of the risk factor(s) are greater than the units of the protective factor(s). This model can be used to describe, in simple terms, causal inferences in complex situations with multiple known and unknown risk and protective factors. It can also justify how people with a low level of exposure to one or more risk factor(s) may be affected by a certain disease while others with a higher level of exposure to the same risk factor(s) may remain unaffected.

## INTRODUCTION

Determining the causality of observed associations is crucial to the use of epidemiologic findings [1]. Causality or causation is a relationship between a process (the cause) and a state (the effect) [2] in which the cause is responsible for the effect, and the effect is dependent on the cause. In general, causality is not simple; instead, it is a complex process involving many known and unknown intermediate factors [3].

The definition of causal criteria was spearheaded by Koch [4] in 1884 as part of his efforts to establish a causal relationship between biological agents and infectious diseases. Building on (and contrasting with) Koch’s criteria, which focused on single causative agents, the concept of a “web of causation” was proposed by MacMahon et al. [5] to emphasize the importance of multiple interrelated levels of disease causation. Indeed, in the context of disease, multiple causes provide better explanations of observed phenomena than do single causes, as no event, condition, or characteristic alone is sufficient to produce disease [3]. As described by Gordis [6], it is nearly impossible to find a one-to-one causal relationship between a risk factor and a disease outcome. In such cases, a necessary and sufficient relationship must exist between the risk factor and the outcome.

Greenland & Brumback [7] categorized causal models into 4 major types: (1) graphical models (causal diagrams), (2) potential-outcome (counterfactual) models, (3) sufficient-component cause (SCC) models, and (4) structural equation models.

Causal diagrams [8-10] provide a framework for causal inference by way of illustrations containing sets of nodes and edges. Nodes represent the variables, while edges represent connections or relationships between variables. As such, a causal diagram is a visualization of the relationships between the variables in a system.

Potential-outcome (counterfactual) models [3,11,12] focus on what would happen to individuals or populations under alternative possible patterns of intervention or exposure. In these models, at least 1 defining condition of the effect must be contrary to fact. In essence, a counterfactual model describes what would have happened if the exposure had been something other than what it was [7].

The SCC model, which was proposed by Rothman et al. [3], is often depicted as a pie chart representing causal inference. This pie chart includes a constellation of risk factors, termed component causes, which act together to form a sufficient cause. Under this framework, a sufficient cause is the minimum combination of conditions and events necessary to inevitably produce disease. These conditions or events can act either simultaneously or sequentially.

Finally, structural equation modeling (SEM) [13] involves a variety of mathematical models, statistical methods, and computer algorithms used to fit construct networks to data. SEM produces a network of causation that is modeled through a system of equations and independence assumptions [7].

These causal models have benefits and limitations. Their focus is on a constellation of predisposing factors that ultimately may lead to the outcome of interest. Indeed, existing causality models have attempted to explain how various intermediate factors are interrelated and contribute to the development of disease. However, the contribution of protective factors, which play an opposing role—that is, they act to prevent the development of the disease—has been neglected. A causal network should take into account both risk factors and protective factors. To the extent that risk factors contribute to disease development, protective factors also play a role in disease prevention. One apparent paradox of causal inference that has remained unanswered is why some people who strongly exhibit a particular risk factor (e.g., heavy smokers) do not develop lung cancer, while others with lower levels of exposure or who lack the risk factor entirely (e.g., non-smokers) do develop it. In this paper, we introduce a new model termed the equivalence model to describe causal inference in an alternative way that addresses the above dilemma. This model, for the first time, creates a place in the causal network for protective factors, illustrating the role of these factors in preventing disease. The equivalence model describes how the imbalance (or balance) between risk factors and protective factors ultimately predicts whether disease development will occur.

## MATERIALS AND METHODS

In a simple, graphical fashion, the equivalence model describes the overall effect of risk factors and protective factors at the individual level. Several risk factors may facilitate the occurrence of the outcome (here, a disease) of interest, while several protective factors may inhibit the development of the same disease. The equivalence model explains how the overall effect predicts the occurrence, or lack thereof, of the outcome.

As a very simple example, suppose that a hypothetical outcome (e.g., hypertension) is affected by only a single risk factor (e.g., salt intake) and a single protective factor (e.g., physical activity [PA]). The risk factor promotes the occurrence of the outcome, while the protective factor suppresses this occurrence. If the risk factor overcomes the protective factor (risk factor > protective factor), the outcome will occur. If the protective factor overcomes the risk factor (risk factor < protective factor), or if a balance exists between the risk and protective factors (risk factor = protective factor), the outcome will not occur. Therefore, the occurrence of the outcome is the consequence of the interplay between the risk factor and the protective factor. This interplay depends on the strength of the association as well as the level and the duration of exposure for each factor. For simplicity, the strength of the association, the level of exposure, and the duration of exposure are classified together under the general term “units” of exposure.

The strength of the association can be measured by the risk ratio (RR) or odds ratio. The stronger the association, the higher the likelihood that the relationship is causal [6]. The level and duration of exposure can be measured using dose-response analysis (e.g., cigarette pack-years). As the dose of positive exposure (that is, exposure to a risk factor) increases, the risk of disease similarly increases [6]. In contrast, the risk of disease decreases as the dose of negative exposure (that is, exposure to a protective factor) increases. For these measurements, comprehensive meta-analyses are required for each risk or protective factor. Although such analysis is possible for known factors, it would be difficult and time-consuming in practice.

As a hypothetical example, suppose the strength of the association between a positive exposure (e.g., cigarette smoking) and a disease (e.g., lung cancer) is 2 (RR = 2), and the level and duration of cigarette smoking for a certain smoker is 10 pack-years. The total number of units of exposure to cigarettes for this person is 2 × 10 = 20. On the opposing side, suppose the strength of the association between a negative exposure (e.g., PA) and lung cancer is 0.5 (RR = 0.5), and the level and duration of PA for the same person is 10 years of moderate-intensity PA. The total number of units of exposure to PA for this person is therefore 0.5/10 = 0.05, the reciprocal of which is 1/0.05, or 20. This person will not develop lung cancer because the units of exposure to the risk factor are equal to the units of exposure to the protective factor (20 = 20). However, if this person increases his or her rate of smoking or ceases to engage in moderate-intensity PA, the balance between risk and protective factors will shift in favor of the risk factors, and he or she will develop lung cancer.

We can represent this relationship with the following equation:

$$E\;=\;\frac{R_{1}\;+R_{2}\;+\;R_{3}\;+\;\cdots \;}{P_{1}\;+\;P_{2}\;+\;P_{3}\;+\;\cdots }$$

Here, E denotes the equivalence variable, *R*_n_ denotes the risk factors, and *P*_n_ denotes the protective factors. When E is less than or equal to 1 (E ≤ 1), the outcome (disease) of interest will not occur. When E is greater than 1 (E > 1), the outcome (disease) of interest will occur.

This equation explains why a subject with greater exposure to a risk factor for a particular disease (e.g., 5 units of exposure) may not develop the disease, while a subject with lower exposure to the same risk factor (e.g., 3 units of exposure) may develop it. An approach limited to risk factors involves focusing solely on the numerator of the equation and ignoring the denominator, which is the part of the equation that involves the protective factors. This introduces confusion, as it fails to explain how the low-risk person could develop the disease while the high-risk person does not. Incorporating the denominator of the equation resolves this confusion. For example, the denominator may be 6 for the first person and 2 for the second. Therefore, the result of the equation for the first person would be E = 5/6 = 0.83 (E < 1), while that of the second person would be E = 3/2 = 1.50 (E > 1). As such, this equation explains in simple terms why a low-risk person may develop a disease, while a high-risk person may remain disease-free.

## RESULTS

**Figure 1.**
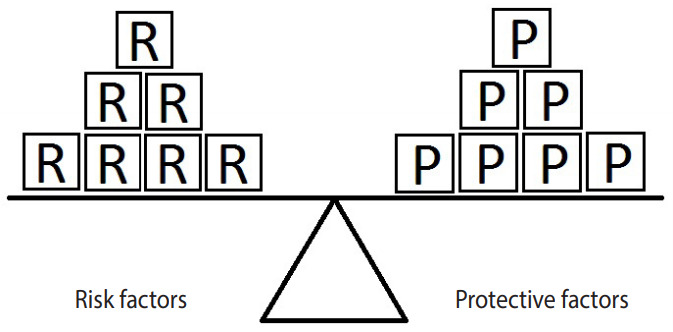
Equivalence model for a hypothetical single risk factor R and a hypothetical single protective factor P. The number of shapes (squares) denotes the units of exposure. A balance exists between the effects of the risk and the protective factors.

Figure 1 represents the simplest form of the equivalence model for a hypothetical disease (e.g., hypertension) affected by a single risk factor (e.g., salt intake) and a single protective factor (e.g., PA). Here, a balance exists between the units of exposure for the risk and protective factors. Neither of the factors can overcome the other; therefore, the outcome will not occur.

**Figure 2.**
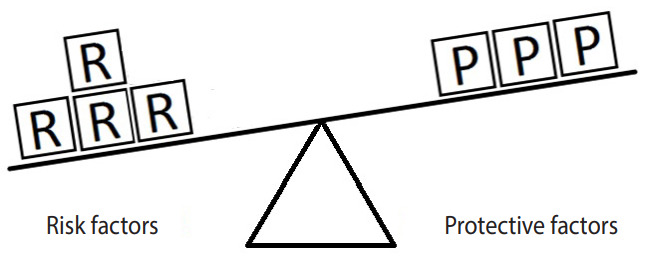
Equivalence model for a hypothetical single risk factor R and a hypothetical single protective factor P. The effect of the protective factor overcomes the effect of the risk factor.

Figure 2 represents an example in which the units of exposure for the risk factor exceed those for the protective factor. In such a situation, the outcome (e.g., hypertension) will occur. In this example, there are 4 units of the risk factor (salt intake) and only 3 units of the protective factor (PA). Therefore, the units of the risk factor overcome the units of the protective factor.

**Figure 3.**
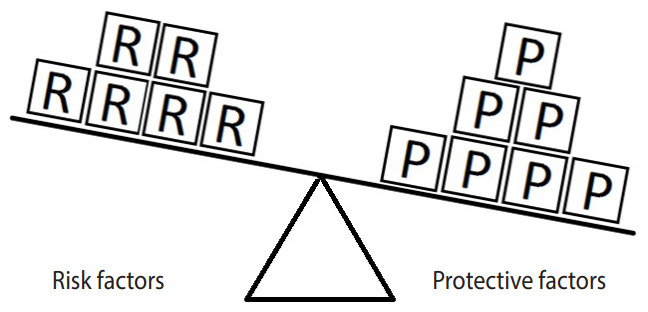
Equivalence model for a hypothetical single risk factor R and a hypothetical single protective factor P. The effect of the risk factor overcomes the effect of the protective factor.

Figure 3 represents the same hypothetical example, except the units of exposure for the risk factor are exceeded by those for the protective factor. In such a situation, the outcome will not occur. Here, there are 7 units of the protective factor compared to 6 units of the risk factor. Since the units of the protective factor overcome the units of the risk factor, the outcome of interest will not occur. This model explains in simple terms why a person with (for instance) exposure to 4 units of salt intake may have high blood pressure (hypertension) while another person with exposure to 6 units of salt intake may not. In particular, the development of a disease depends on the levels of both risk and protective factors. Therefore, a person who engages in moderate to vigorous PA and whose daily salt intake is high may have normal blood pressure, whereas another person who engages in mild PA and whose daily salt intake is low may have high blood pressure (hypertension).

The equivalence model can also represent more complex situations. As previously mentioned, it is nearly impossible for a single factor to serve as both a necessary and a sufficient cause of the development of a disease [6]. Therefore, diseases are typically the consequence of the interplay between multiple known and unknown risk factors and protective factors.

**Figure 4.**
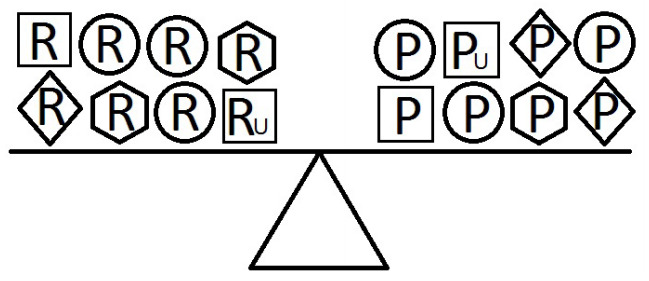
Equivalence model for multiple risk and protective factors. Risk factors are denoted by R and protective factors by P. Unknown risk and protective factors are denoted by R_U_ and P_U_, respectively. Different shapes (circle, square, diamond, or hexagon) denote different risk or protective factors. The number of shapes represents the number of units of exposure. A balance exists between the overall effect of the risk factors and the overall effect of the protective factors.

**Figure 5.**
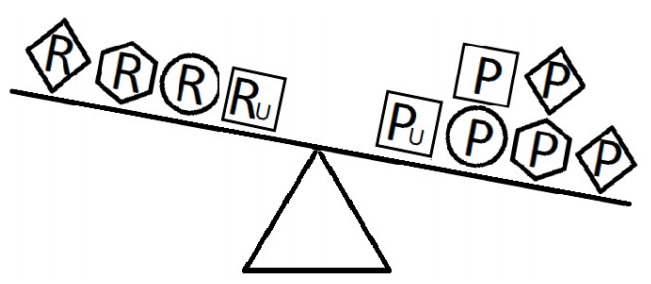
Equivalence model for multiple risk and protective factors. Risk factors are denoted by R and protective factors by P. Unknown risk and protective factors are denoted by R_U_ and P_U_, respectively. Different shapes (circle, square, diamond, or hexagon) denote different risk or protective factors. The number of shapes represents the number of units of exposure. The overall effect of the protective factors overcomes the overall effect of the risk factors.

**Figure 6.**
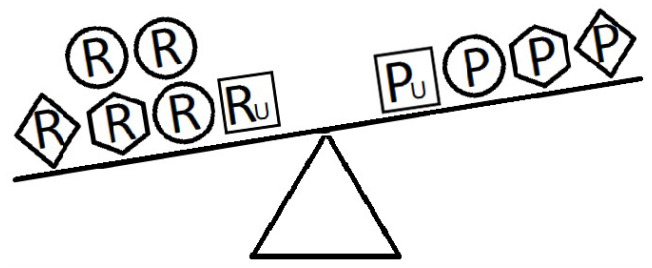
Equivalence model for multiple risk and protective factors. Risk factors are denoted by R and protective factors by P. Unknown risk and protective factors are denoted by R_U_ and P_U_, respectively. Different shapes (circle, square, diamond, or hexagon) denote different risk or protective factors. The number of shapes represents the number of units of exposure. The overall effect of the risk factors overcomes the overall effect of the protective factors.

Figures 4-6 indicate a complicated interplay between risk and protective factors with regard to the development of the disease of interest. As previously articulated, the risk factors increase the probability of disease development, while the protective factors reduce this probability.

The strength of the association between various risk and protective factors and the outcome (disease) of interest can vary. The level and duration of exposure for both risk and protective factors also vary from person to person. As mentioned in the Methods section, the units of exposure refer to the combination of the strength of the association, the level of exposure, and the duration of exposure.

When a balance exists between the units of exposure of the risk and protective factors, as shown in Figure 4, the overall effect of the known and unknown risk factors is equal to the overall effect of the known and unknown protective factors. Therefore, the disease will not develop (E = 1). A similar outcome occurs when the units of the protective factors exceed those of the risk factors (E < 1), as shown in Figure 5. In such a situation, the overall effect of the known and unknown protective factors overcomes the overall effect of the known and unknown risk factors, and the disease will thus be avoided. In contrast, if the units of the risk factors exceed those of the protective factors (E > 1), as shown in Figure 6, the disease will develop, because the overall effect of the known and unknown risk factors overcomes the overall effect of the known and unknown protective factors.

## DISCUSSION

In a simple fashion, the equivalence model can justify why (for example) a moderate smoker or even a non-smoker may be affected by lung cancer while a heavy smoker may remain unaffected. The crucial relationship is the balance between the overall effect of known and unknown risk factors and that of known and unknown protective factors. If the overall effect of the risk factors is greater than the overall effect of the protective factors, then the disease (e.g., lung cancer) will develop. However, if the overall effect of the protective factors is greater than that of the risk factors, disease development will not occur.

The case of Fidel Alejandro Castro, the former leader of the Republic of Cuba, illustrates the benefit of the equivalence model over existing causal models in the interpretation of the causal relationship between smoking and lung cancer. Castro smoked for more than a half-century, but his death was unrelated to lung cancer, while many light smokers or even non-smokers die from lung cancer. The existing causal models would explain this issue in different ways. The SCC model would posit that the component causes, which act in concert, were insufficient to produce lung cancer. SEM analysis would predict that mathematical modeling, algorithms, and statistical methodology fail to align the data involved in this situation with networks of constructs associated with the development of lung cancer. The concept of the web of causation would posit that smoking alone is not enough, and other causes in the network must be present for lung cancer to develop. Finally, the counterfactual model would attempt to explain this situation by constructing a hypothetical scenario in which at least 1 condition in the causal pathway was contrary to fact and by then attempting to predict the effect. All of these causal models focus on the constellation of risk factors that act together to promote the development of disease. These models do not—at least directly and explicitly—consider the opposite role of protective factors. In contrast, according to the equivalence model, it is the balance between risk and protective factors that determines whether lung cancer will develop. Per this model, Castro (despite being a heavy smoker for many years) did not develop lung cancer because he may have been shielded by known and unknown protective factors that neutralized the effect of smoking on lung cancer.

The equivalence model accommodates both combined and independent causal mechanisms for the same outcome. As such, any subject may simultaneously exhibit multiple risk and protective factors to varying extents. Each factor has an effect on the outcome of interest. Therefore, an interplay exists between risk factors and protective factors in terms of severity, level, and duration of exposure. In a causal network, factors may have synergistic or antagonistic effects on one another. However, the overall effect of risk factors and protective factors determines whether the outcome of interest occurs. If the overall effect of the risk factors overcomes the overall effect of the protective factors, the outcome will occur; otherwise, it will not. This model can not only be used to describe the complex process of chronic disease development in the presence or absence of a wide variety of known risk and protective factors, but it can also be applied to acute conditions such as infectious diseases.

The SCC model, or Rothman’s pie, explains that an outcome will occur if and only if 2 or more factors together are necessary and sufficient [3,7,14]. According to this model, several known and unknown factors termed component causes act simultaneously or sequentially to form a sufficient cause. Therefore, the presence of several component causes will not result in the occurrence of the outcome until a sufficient cause is formed. However, the SCC model does not explain why people with a lower dose of exposure to a particular risk factor (or who lack the risk factor entirely) may develop a disease, while other people who strongly exhibit that risk factor may be unaffected. The equivalence model is a simple method that explains this seemingly paradoxical phenomenon.

The equivalence model has a few limitations. First, this model provides causal inferences qualitatively rather than quantitatively. Although measuring the level, duration, and intensity of exposure (which, in combination, we have termed the unit[s] of exposure) of known risk and protective factors is possible by performing dose-response meta-analyses, it is very complicated and excessively time-consuming. Second, for the simplicity of the model, the synergistic and antagonistic interactions among risk and protective factors were not taken into account, as measuring such interactions is extremely complicated and may even be impossible. Third, the role of unknown risk and protective factors is another challenge for this model and, of course, for other existing causal models as well.

## CONCLUSION

This model is a simple method to describe causal inferences in complicated situations with multiple known and unknown risk and protective factors. Moreover, it can explain why a group of people who lack or have low exposure to one or more risk factor(s) may develop a disease, while another group of people who strongly exhibit the same risk factor(s) may remain unaffected. This model can be applied to both acute and chronic conditions.
